# Her-2/*neu-*triggered intracellular tyrosine kinase activation: *in vivo* relevance of ligand-independent activation mechanisms and impact upon the efficacy of trastuzumab-based treatment

**DOI:** 10.1038/sj.bjc.6601160

**Published:** 2003-09-09

**Authors:** G Hudelist, W J Köstler, J Attems, K Czerwenka, R Müller, M Manavi, G G Steger, E Kubista, C C Zielinski, C F Singer

**Affiliations:** 1 Clinical Division of Special Gynaecology, Department of Obstetrics and Gynaecology, and Center for Excellence in Clinical and Experimental Oncology, University Hospital, Vienna, Austria; 2 Clinical Division of Oncology, Department of Medicine I, and Center for Excellence in Clinical and Experimental Oncology, University Hospital, Vienna, Austria; 3 Department of Pathology, Otto Wagner Hospital, Vienna, Austria; 4 Division of Gynaecopathology, Department of Pathology, and Center for Excellence in Clinical and Experimental Oncology, University Hospital, Vienna, Austria; 5 Ludwig Boltzmann Institute for Clinical-Experimental Oncology, Vienna, Austria

**Keywords:** Her-2/*neu*, tyrosine kinase phosphorylation, breast cancer, trastuzumab

## Abstract

Proteolytic cleavage of the Her-2/*neu* extracellular domain (ECD) has been shown to initiate receptor phosphorylation representing Her-2/*neu* activation *in vitro*. The present investigation was performed to evaluate the clinical relevance of ECD cleavage for Her-2/*neu* activation and the consequences of active intracellular Her-2/*neu* signalling reflected by tyrosine kinase phosphorylation in patients treated with the anti-Her-2/*neu* antibody trastuzumab. Sera from 62 patients receiving trastuzumab-based treatment for Her-2/*neu* overexpressing metastatic breast cancer were assessed for pretreatment ECD levels using an enzyme-linked immunosorbent assay. In parallel, Her-2/*neu* activation status of tumour specimens was assessed by immunohistochemistry using a Her-2/*neu* phosphorylation state specific antibody (PN2A) and correlated with the patients’ ECD levels and clinical course of disease. Serum ECD levels were significantly higher in 15 (24%) patients with tumours exhibiting activated Her-2/*neu* as compared to those without detectable Her-2/*neu* phosphorylation (median 148.2 *vs* 28.5 ng ml^−1^, *P*=0.010). Whereas response rate only showed a trend to be higher in patients with Her-2/*neu*-phosphorylated breast cancer (47 *vs* 34%, *P*=0.197), both uni- and multivariate analyses revealed that the median progression-free survival under trastuzumab-based treatment was significantly longer in patients with Her-2/*neu*-phosphorylated breast cancer–11.7 (95% CI 5.2–18.3) months–when compared to the progression-free survival of 4.5 (95% CI 3.4–5.6) months observed in patients with tumours lacking phosphorylated Her-2/*neu* (*P*=0.001). Proteolytic cleavage of the ECD represents a biologically relevant ligand-independent mechanism of Her-2/*neu* activation *in vivo*. The influence of Her-2/*neu* activation status upon the outcome of trastuzumab-based therapies merits further investigation in larger prospective trials.

The human epidermal growth factor (Her-2/*neu*) is a 185 kDa oncoprotein (p185), which is overexpressed in 25–30% of invasive breast cancers (Schechter *et al*, 1984; Slamon *et al*, 1987; Olayioye *et al*, 2000). Her-2/*neu* overexpression has consistently been found to confer resistance to cytotoxic and endocrine therapy and to account for an aggressive biological behaviour, thereby resulting in shorter disease-free and overall survival in both, patients with early and advanced breast cancer (Slamon *et al*, 1987).

The Her-2/*neu* molecule is composed of an extracellular ligand-binding domain, an amphipathic transmembrane region and an intracellular tyrosine kinase domain, which contains a carboxy tail with five major autophosphorylation sites (Ullrich and Schlessinger, 1990). Ligand binding is thought to initiate the formation of homo- and heterodimeric receptor complexes with other growth factor receptor (GFR) class 1 family members such as EGFR, Her-3 and Her-4 into which Her-2/*neu* is recruited as a preferential dimerisation partner (Tzahar *et al*, 1996). This process is followed by intrinsic tyrosine kinase-mediated autophosphorylation and mutual phosphorylation of the respective dimerisation partners and ultimately results in activated receptor complexes (Segatto *et al*, 1990; Tzahar *et al*, 1996). Alternatively, *in vitro* Her-2/*neu* activation has also been demonstrated to occur as a consequence of spontaneous cleavage of its extracellular domain (ECD) thereby resulting in the production of a truncated membrane-bound fragment (p95) with kinase activity (Christianson *et al*, 1998; Molina *et al*, 2001). Since the p95 fragment has also been detected in breast cancer specimens, it has been suggested that shedding of the ECD may represent an alternative activation mechanism of Her-2/*neu* *in vivo* (Christianson *et al*, 1998).

Intracytoplasmatic phosphorylated tyrosine residues of the Her-2/*neu* molecule function as high-affinity binding sites for SH_2_ domain containing proteins, which link the receptor to intracellular signal transduction processes such as the *ras-raf*-mitogen activated protein kinase (MAPK) and the phosphatidylinositol-3 kinase (PI-3 K) pathways. Both are believed to be key elements in the regulation of cell proliferation and survival (reviewed in Slichenmyer and Fry, 2001). In this context, phosphorylation of the 1248 tyrosine residue (Tyr1248), which is supposed to constitute the main autophosphorylation site of Her-2/*neu*, is a key event for downstream signalling (Akiyama *et al*, 1991; Dougall *et al*, 1994; DiGiovanna and Stern, 1995; Marshall, 1995).

Monoclonal antibodies targeting the Her-2/*neu* ectodomain, such as trastuzumab and its murine precursor 4D5, have been shown to abrogate the Her-2/*neu* activation processes and to interfere with Her-2/*neu*-dependent gene expression, to modulate cell cycle progression, induce cellular differentiation and to sensitise Her-2/*neu* overexpressing cells to apoptotic stimuli. These effects are mediated by several distinct mechanisms including the blockade of ligand binding, disruption of homo- and heterodimer formation, induction of receptor internalisation and degradation as well as prevention of cleavage of the ECD (Baselga *et al*, 2001; Yip and Ward, 2002), all of which ultimately result in a decrease of receptor phosphorylation *in vitro* (Kumar *et al*, 1991; Lane *et al*, 2000). An intact tyrosine kinase activity appears to be the main precondition for growth inhibition induced by trastuzumab, since the regular functions of all of the mentioned mechanisms depend upon Her-2/*neu* kinase integrity (Xu *et al*, 1994).

These observations have been successfully translated into clinical use. Thus, trastuzumab has not only demonstrated activity as a single agent, but had also exerted synergistic activity when administered in conjunction with cytotoxic drugs, thus resulting in prolonged progression-free and overall survival in patients with Her-2/*neu* overexpressing metastatic breast cancer (Slamon *et al*, 2001; Vogel *et al*, 2002). Currently, the decision for treating patients with metastatic breast cancer with trastuzumab is based upon the detection of Her-2/*neu* overexpression and/or amplification of the *c-erbB-2* gene determined by immunohistochemistry (IHC) and fluorescence *in situ* hybridisation (FISH), respectively. However, aside from the intensity of Her-2/*neu* overexpression, additional factors that would predict the effectivity of trastuzumab as single agent or in combination with cytotoxic treatment are still lacking. Since tyrosine kinase activation is the downstream mechanism of action for Her-2/*neu*, it has been postulated that tumours exhibiting active Her-2/*neu* signalling represented by phosphorylation of tyrosine residues might be those most sensitive to treatment with trastuzumab (DiGiovanna and Stern, 1995; Thor *et al*, 2000).

The aim of the present study was to determine whether cleavage of the Her-2/*neu* ECD is correlated with overall tyrosine kinase activity *in vivo* and might therefore constitute a clinically relevant ligand-independent mechanism for the activation of Her-2/*neu* in breast cancer. In addition, we correlated the clinical course of disease of patients treated with trastuzumab with Her-2/*neu* phosphorylation status to determine the clinical relevance of active Her-2/*neu* signalling.

## MATERIALS AND METHODS

### Patient population–inclusion and exclusion criteria

Patients who had received trastuzumab (Herceptin®, Roche Pharmaceuticals, Vienna, Austria)±chemotherapy at our institution between April 2000 and February 2003 in accordance with previously published treatment protocols (Pegram *et al*, 1998; Cobleigh *et al*, 1999; Burstein *et al*, 2001; Miller *et al*, 2001; Seidman *et al*, 2001; Slamon *et al*, 2001; Esteva *et al*, 2002; Vogel *et al*, 2002) were identified retrospectively by using pharmacy protocols. Trastuzumab was administered as 4 mg kg^−1^ loading dose followed by a weekly 2 mg kg^−1^ maintainance dose, as described in Cobleigh *et al* (1999), to patients with metastatic breast cancer with grade 2+ or 3+ Her-2/*neu* overexpression assessed by immunohistochemistry. Patients included into the present analysis were required to have bidimensionally measurable (with both diameters >1.0 cm and at least one lesion with both diameters >1.5 cm) disease (excluding previously irradiated or bone lesions as the only site of measurable disease) with clearly defined margins and radiologically (CT and/or MRI and/or ultrasound) documented tumour progression before initiation of trastuzumab-based treatment.

In addition, patients’ records were required to contain documented response assessment performed every 6–8 weeks (depending on the therapeutic regimen). Response evaluation was performed by independent review of patients records and radiology reports by two investigators and classified in accordance with the Southwest Oncology Group response criteria and end point definitions (Green and Weiss, 1992). Patients who had discontinued treatment before radiological response assessment or had been lost to follow-up (as defined by the last record obtained >8 weeks before the present analysis) were censored as therapeutic failures or deaths, respectively. Patients who had previously received treatment with monoclonal antibodies, vaccines or biological response modifiers were excluded. Further inclusion criteria consisted of availability of intact paraffin-embedded tissue (excluding mechanically altered tissue and fine needle aspirates) from which the original assessment of Her-2/*neu* overexpression had been performed and deep-frozen (−80°C) sera that had been obtained immediately before the first infusion of trastuzumab. In accordance with our institutional ethical committee guidelines, signed informed consent was obtained from all patients before 8 ml of blood was drawn from the same venous access, which was afterwards immediately used for infusion of trastuzumab. Data on oestrogen and progesterone receptor status of tissue samples from which the original assessment of Her-2/*neu* overexpression had been performed were available from pathology records.

### Determination of Her-2/*neu* overexpression

In all cases, reassessment of Her-2/*neu* overexpression and determination of Her-2/*neu* phosphorylation were performed independently by two experienced pathologists blinded to the clinical course of patients and the results of other tests performed. All testing was performed in the identical tissue material used for initial patient selection for trastuzumab therapy. Her-2/*neu* protein expression was evaluated on paraffin-embedded tissue using the HercepTest kit (DAKO A/S, Glostrup, Denmark) for immunoenzymatic staining in accordance with the protocol described in the manufacturer's guide: tissue sections were dewaxed in xylene and then rehydrated through ethanol to distilled water. Subsequently, tissue sections were immersed in epitope retrieval solution (DAKO) at 95°C, and then in waterbath at 95°C for a total of 40 min, followed by a 20 min cool-down period at room temperature. Slides were incubated at room temperature with the primary rabbit polyclonal antibody to the Her-2/*neu* oncoprotein (supplied by the kit manufacturer) on a DAKO Autostainer for 30 min followed by application of peroxidase-blocking reagent. Antibody was localised by incubating slides with the DAKO Visualization Reagent using horseradish peroxidase-conjugated goat anti-rabbit immunoglobulins for 30 min using the DAKO Autostainer. Sections were finally incubated with diaminobenzidine (DAB) as chromogen and counterstained with haematoxylin. To assess the expression of the Her-2/*neu* oncoprotein accurately, positive controls consisting of freshly cut breast cancer cases known to overexpress Her-2/*neu* and a control slide consisting of three pelleted, formalin-fixed, paraffin-embedded human breast cell lines with staining intensity scores of 0, 1+ and 3+ (supplied by the kit manufacturer) were included in each staining run. Negative controls were performed by substitution of the HER-2/*neu* primary antibody by normal rabbit serum (DAKO Negative Control Reagent). Only membrane staining intensity and pattern were evaluated using the 0 to 3+ scale as illustrated in the HercepTest kit scoring guidelines.

### Determination of Her-2/*neu* gene amplification

In cases of 2+ immunostaining for Her-2/*neu*, oncoprotein gene copy number of the HER-2/*neu* (c-*erb*B-2) gene was determined by dual-colour FISH referring to the numbering of chromosome 17 using the PathVysion® HER-2 DNA probe-kit (Vysis Inc., Downers Grove, IL, USA) according to the manufacturer's directions. The HER2/*neu*-SpectrumOrange probe contains a DNA sequence specific for the *c-erbB-2* human gene locus and hybridises to region 17q11.2–q12 of human chromosome 17. The CEP 17 (chromosome enumeration probe 17)/SpectrumGreen probe contains alpha-satellite DNA that hybridises to the D17Z1 locus (centromere region of chromosome 17). Fluorescence was evaluated by using a Nikon E600 microscope with a Y-Fl Epi-Fluorescence Attachment (Nikon, Tokyo, Japan) and a black-and-white, charge-coupled device (CCD) camera (COHU 4912; Cohu, San Diego, CA, USA) run by Lucia-Fish software (Laboratory-Imaging, Prague, Czech Republic). Fluorescence *in situ* hybridisation slides were compared with adjacent haematoxylin/eosin slides. Her-2/*neu* signals and CEP17 signals in at least 20 cancer nuclei per tumour were scored. A cell was considered amplification positive if the Her2/*neu* : CEP17 ratio exceeded 2.

### Determination of specifity of phospho-specific (P-Tyr1248) Her-2/*neu* antibody (PN2A)

Her-2/*neu* (p185) overexpressing SKBR3 human mammary carcinoma cells (American Type Culture Collection, Rockville, MD, USA) were cultured in McCoy's 5A modified medium (Gibco BRL, Paisley, Scotland), supplemented with 15% of heat-inactivated fetal calf serum (FCS), glutamine and 50 IU penicillin and 50 *μ*g ml^−1^ streptomycin (all Gibco) in a humidified atmosphere containing 5% CO_2_. Confluent SKBR3 cells were cultivated in six-well plates (Corning Inc., NY, USA) and serum starved in medium containing 0.1% FCS overnight. Since epidermal growth factor (EGF) has been shown to stimulate tyrosine phosphorylation of Her-2/*neu* dramatically (DiGiovanna and Stern, 1995), cells were then incubated with and without EGF (human rEGF, Strathmann Biotech AG, Hamburg, Germany) at 100 ng ml^−1^ for 2, 4, 8, 10, 30 and 60 min at room temperature. To maximise differential phosphorylation of p185 isolated from EGF-treated cells, the phosphotyrosine phosphatase inhibitor sodium ortho-vanadate (Alexis Biochemicals, QBIOGENE Inc., Carlsbad, CA, USA) was added at a final concentration of 500 μM during the final hour prior to stimulation of cultures that were to be stimulated with EGF. After incubation, cells were lysed in 0.5 ml 1 × Laemli buffer and boiled at 96°C for 10 min.

To perform Western blotting, cell lysates were separated on a 7.5% SDS–polyacrylamide electrophoretic gel (PAGE) with a constant voltage of 200 V for 30 min and transferred to a nitrocellulose membrane (Novex) by electroblotting (20 V, 1 h). SeeBlue® Pre-stained Standards (250, 98 and 64 kDa) (Novex, Invitrogen, Paislay, UK) were used as molecular weight standards. Membranes were incubated in blocking solution for 1 h at room temperature and treated afterwards with 1 *μ*g ml^−1^ of mouse monoclonal antibody: (A) mouse monoclonal c-erbB-2/HER-2/neu Ab-20 (L87+2ERB19), (B) c-erbB-2/HER-2/neu (phospho-specific) Ab-18 (clone PN2A, both NeoMarkers, Fremont, CA, USA) at room temperature for 2 h. Membranes were incubated in alkaline phosphatase-coupled anti-mouse antibody for 1 h at room temperature after a 30 min wash. Following another washing, the membranes were incubated in 4-nitro blue tetrazolium chloride/5-bromo-4-chloro-3-indolyl phosphate (NBT/BCIP, Roche Diagnostics GmbH, Vienna, Austria) developer solution for 10 min. Membranes were developed and finally washed with bidistilled water.

### Determination of Tyr1248 Her-2/*neu* phosphorylation of tumour samples

Determination of Her-2/*neu* phosphorylation was performed using the monoclonal antibody PN2A (NeoMarkers, Westinghouse Drive, Fremont, CA, USA). As demonstrated by Western blotting analysis (see above and Green and Weiss, 1992) and immunohistochemistry using peptide blocking experiments (Green and Weiss, 1992) the PN2A antibody specifically recognises phosphorylation of the major autophosphorylation site Tyr-1248 (P-Tyr1248) without crossreactivity with c-erbB-1 (EGFR), c-erbB-3 or c-erbB-4, or unphosphorylated Her-2/*neu*. Formalin-fixed paraffin-embedded tissue sections (4 *μ*m) were deparaffinised, rehydrated, and endogenous peroxidase-blocked with 2% hydrogen peroxide. Antigen retrieval was performed by placing sections in 10 mmol l^−1^ citrate buffer (pH 6.0) and microwave treatment for 15 min. Slides were allowed to cool to room temperature (RT), washed with phosphate-buffered saline (PBS) and distilled water, and blocked with Ultra V Block (Lab Vision, Westinghouse Drive, Fremont, CA, USA). PN2A (6 *μ*g ml^−1^) was applied and sections were incubated at 4°C overnight. After two additional PBS washes, sections were sequentially incubated at RT for 30 min with biotinylated goat anti-polyvalent (Lab Vision, Westinghouse Drive, Fremont, CA, USA) and streptavidin-HRP (Lab Vision, Westinghouse Drive, Fremont, CA, USA). Subsequently, slides were incubated with 3-amino-9-ethylcarbazole (AEC, a widely used chromogen), counterstained with haematoxylin and cover-slipped.

**Figure 1:**
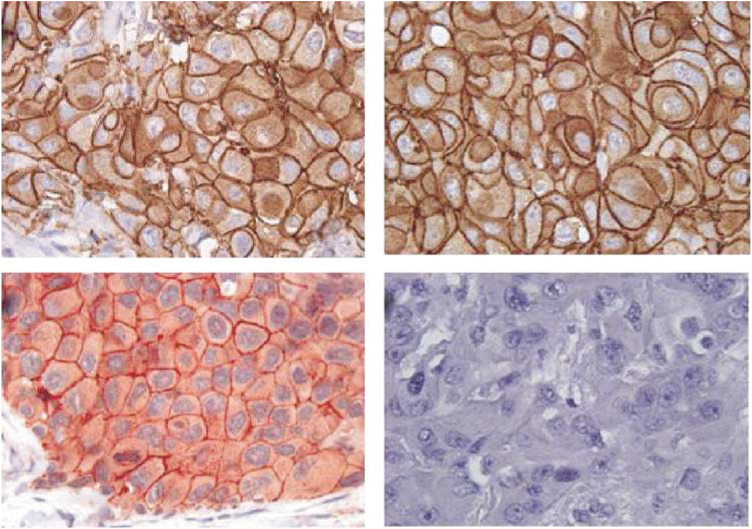
Immunohistochemical analysis of two (grade 3+) Her-2/*neu* overexpressing tumours (upper row) differing with respect to Her-2/*neu* phosphorylation status at the tyr1248 residue (lower row): strong (left) and absent Her-2/*neu* phosphorylation (right).

Expression of phosphorylated Her-2/*neu* was visually assessed using the same scoring system applied for determination of Her-2/*neu* overexpression (see above). In contrast to evaluation of receptor overexpression of Her-2/*neu* (considering grade 2+ and grade 3+tumours positive), tumours exhibiting a clearly discernible positive signal for receptor phosphorylation (⩾grade 1+using Herceptest guidelines) on cellular membranes were considered positive, because even weak staining for phosphorylation of the 1248 tyrosine residue of the Her-2/*neu* molecule (pHER-2/*neu*) might represent tyrosine kinase activity, that is, active receptor signalling. In contrast, faint cytoplasmatic staining in the absence of membraneous staining was not considered positive. For each assay, pelleted, formalin-fixed, paraffin-embedded human T47D and rhEGF-stimulated SKBR-3 breast cancer cell lines (American Type Culture Collection, Rockville, MD, USA) were used as positive controls. Photomicrographs of immunohistochemical analysis for Her-2/*neu* overexpression and Her-2/*neu* phosphorylation are depicted in Figure 1

.

### Determination of serum Her-2/*neu* ECD levels

Sera obtained immediately before the first infusion of trastuzumab were analysed using a Sequential Solid Phase Sandwich Human Her-2/*neu* Quantitative ELISA (Her-2/*neu* Microtiter ELISA, Oncogene Science, Cambridge, MA, USA) according to the manufacturer's instructions. Microplates were washed using an automated washer (Dias Microplate Washer, Dynex Technologies, Denkendorf, Germany). Absorbance reading was performed using an automated reader (FLUOstar Galaxy, BMG Labtechnologies, Offenburg, Germany) and serum ECD concentrations were calculated from absorbance data using the Fluoscan Galaxy software (vers. 4.20-0, BMG Labtechnologies, Offenburg, Germany). Intra- and interassay precision of the assay were always <5% CV.

### Statistical analysis

Frequencies of patients’ characteristics were compared by Fisher's exact test, and differences in Her-2/*neu* ECD levels, age and recurrence-free interval (time from initial diagnosis to appearance of metastatic disease) with Mann–Whitney's *U*-test. After performance of a normalising transformation on serum Her-2/*neu* ECD, a multiple analysis of variance (ANOVA) was assessed using serum Her-2/*neu* ECD as dependent variable. Relationships between serum Her-2/*neu* ECD and the presence of phosphorylation were adjusted by the grade of Her-2/*neu* overexpression, the presence or absence of visceral metastases and the number of sites with metastatic disease. The results of the multiple analysis of variance are represented by medians and 95% confidence intervals (95% CI). For all subsequent analyses, grade of Her-2/*neu* overexpression (2+ *vs* 3+), presence or absence of Tyr1248 phosphorylation, patient age, recurrence-free interval, anthracycline pretreatment (for early breast cancer or metastatic disease), number of prior chemotherapeutic regimens for metastatic disease, oestrogen receptor (ER) and progesterone receptor (PgR) status, Karnofsky's performance index, presence or absence of visceral metastases (liver and/or lung), number of organs involved by metastatic disease, type of treatment (single-agent trastuzumab *vs* combination with chemotherapy) and baseline serum Her-2/*neu* ECD levels were entered as variates. Multiple logistic regression analyses were used to determine whether any of these variates could predict response or clinical benefit (response or disease stabilisation) from trastuzumab-based treatment. In analogy, multiple Cox regression models were utilised to identify the properties of the predictors mentioned above on progression-free and overall survival. Confounders without significant influences were removed by the backward selection method based on the Wald statistic. The related risk (RR), the odds ratio (OR), and 95% CI were calculated with the proportional hazard method in respect of Cox regression and logistic regression. Survival curves (progression-free and overall survival) were compared with the log-rank test. For all analyses, a *P*-value <5% was considered statistically significant. SPSS statistical software system (SPSS Inc., Chicago, IL, USA, version 10.0) was used for all calculations.

## RESULTS

### Study population

A total of 69 patients with Her-2/*neu* overexpressing metastatic breast cancer, who received trastuzumab-based treatment at our institution during the indicated period, were identified. Seven patients were excluded for the present analysis due to the following reasons: nonavailability of tissue samples from which determination of Her-2/*neu* overexpression had been performed (one patient), mechanically altered tissue samples not amenable for subsequent testing (one patient), nonavailability of sera obtained immediately before the first infusion of trastuzumab (five patients). Thus, the final study population comprised 62 patients (median age 52.6, range 27.6–80.9 years). Patients’ characteristics are depicted in accordance to Her-2/*neu* phoshorylation status (pHER-2/*neu*) of tumour samples in Table 1

**Table 1:** Patients and treatment characteristics according to phosphorylation status of Her-2/*neu* (pHer-2/*neu*)

		**pHer-2/*neu* positive (*n*=15)**	**pHer-2/*neu* negative (*n*=47)**	***P*-value**
Median age (range) (years)		52.9 (27.6–73.5)	52.2 (23.4–80.9)	0.616^a^
Her-2/*neu* expression	Grade 2+	3 (20%)	5 (11%)	
	Grade 3+	12 (80%)	42 (89%)	0.388^b^
Oestrogen receptor status	Positive	3 (20%)	17 (36%)	
	Negative	12 (80%)	30 (64%)	0.346^a^
Progesterone receptor status	Positive	3 (20%)	9 (19%)	
	Negative	12 (80%)	38 (81%)	1.000^b^
Histologic type	Ductal	12 (80%)	40 (85%)	
	Other	3 (20%)	7 (15%)	0.693^b^
Grading	1	1 (7%)	1 (2%)	
	2	1 (7%)	9 (19%)	
	3	13 (87%)	37 (79%)	0.384^c^
Sites of active disease	Breast	2 (13%)	8 (17%)	0.545^a^
	Axilla	1 (7%)	7 (15%)	0.667^a^
	Liver	10 (67%)	25 (53%)	0.390^a^
	Lung	5 (33%)	22 (47%)	0.390^a^
	Skin/soft tissue	1 (7%)	10 (21%)	0.268^a^
	Distant lymph nodes	5 (33%)	24 (51%)	0.254^a^
	Bone	9 (60%)	23 (49%)	0.558^a^
	Other	3 (20%)	17 (36%)	0.346^a^
Number of organs affected by metastatic disease	1 or 2	9 (60%)	24 (51%)	
	More	6 (40%)	23 (49%)	0.570^a^
Metastatic disease to visceral organs		13 (87%)	35 (74%)	0.484^a^
Median (range) recurrence-free interval (months)		20.7 (0.0–85.4)	23.0 (0.0–108.6)	0.755^a^
Anthracycline pretreatment (for primary and/or metastatic breast cancer)	Yes	14 (93%)	41 (87%)	
	No	1 (7%)	6 (13%)	1.000^b^
Number of previous chemotherapeutic regimens for metastatic disease	0	11 (73%)	33 (70%)	
	1	1 (7%)	10 (21%)	
	2 or more	3 (20%)	4 (9%)	0.188^c^
Karnofsky's performance status	⩾70%	8 (53%)	26 (55%)	
	< 70%	7 (47%)	21 (45%)	1.000^b^
Treatment	Single-agent trastuzumab			
	(Cobleigh *et al*, 1999)	1 (7%)	5 (11%)	
	Trastuzumab+vinorelbine			
	(Burstein *et al*, 2001)	11 (73%)	24 (51%)	
	Trastuzumab+docetaxel			
	(Esteva *et al*, 2002)	1 (7%)	8 (17%)	
	Trastuzumab+paclitaxel			
	(Seidman *et al*, 2001)	1 (7%)	4 (9%)	
	Trastuzumab+other chemotherapeutic agents (Pegram *et al*, 1998; Miller *et al*, 2001)	1 (7%)	6 (13%)	0.654^b^

a Mann–Whitney *U*-test.

b Fisher's exact test (two-sided).

c Pearson's *χ*^2^.

. This analysis covers a median observation period of 22.4 (range 6.1–37.8) months, during which 19 (31%) objective responses (including eight complete responses) were observed, 19 (31%) patients experienced durable disease stabilisation (⩾4 months) and 24 (39%) of patients had primarily progressive disease. As of February 2003, 55 (89%) patients had experienced disease progression and 28 (45%) deaths had occurred, all of which were attributed to disease progression. No patient was lost to follow-up. Median (95% CI) progression-free and overall survival calculated from survival function were 5.7 (95% CI 2.5–8.9) and 23.8 (95% CI 16.7–30.8) months, respectively.

### Specifity of phospho-specific (P-Tyr1248) Her-2/*neu* antibody (PN2A)

Figure 2

**Figure 2:**
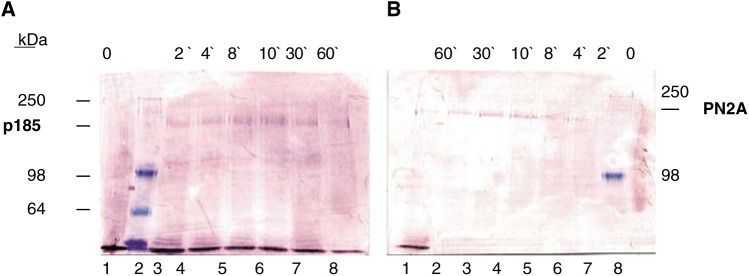
Western blot analysis demonstrating the phosphorylation state specifity of the PN2A antibody. Specifity of (**A**) anti-Her-2/*neu* (p185) antibody and (**B**) anti-phospho (tyr1248) Her-2/*neu* antibody (PN2A) is shown by Western blot analysis of whole-cell lysates. SKBR3 cells were treated without (lanes A1 and B8) and with 100 ng ml^−1^ EGF for 2 (lanes A3 and B6), 4 (lanes A4 and B5), 8 (lanes A5 and B4), 10 (lanes A6 and B3), 30 (lanes A7 and B2) and 60 (lanes A8 and B1) min (kDa, molecular weight in kilodalton, molecular weight markers at 250, 98 and 64 kDa).

shows the results of Western blotting performed on EGF-stimulated SKBR3 cells by using the PN2A (Figure 2B) and the control Her-2/*neu* antibody (Figure 2A). Whereas the Her-2/*neu* antibody detects Her-2/*neu* independent of its activation status, the PN2A antibody specifically recognises the activated, tyrosine phosphorylated (P-Tyr1248) form of Her-2/*neu*. This is supported by enhanced tyrosine kinase activities (larger PN2A bands from left to right until point of saturation) corresponding to different treatment periods of SKBR3 cells with EGF (compare Figure 2B, lanes 8, 6-3).

### Frequencies of Her-2/*neu* gene amplification, protein overexpression and receptor activation

Immunohistochemical analysis for Her-2/*neu* expression demonstrated grade 2+ overexpression in eight (13%) and grade 3+ staining in 54 (87%) of tumour samples. FISH analysis of grade 2+ Her-2/*neu* overexpressing samples did not reveal gene amplification in any of the specimens examined. Positive immunohistochemical staining of the cell membranes for phosphorylation of the Her-2/*neu* 1248 tyrosine residue (tyr1248) was observed in 15 (24%) tissue samples with intense staining detected in four, moderate staining in three and weak, but clearly discernible staining for receptor phosphorylation in eight tissue samples, respectively. Moderate or intense staining for tyr1248 (pHer-2/*neu*) was associated with grade 3+ Her-2/*neu* overexpression in all cases, whereas weak staining for tyr1248 was observed in five grade 3+ and three grade 2+ Her-2/*neu* overexpressing tumours.

### Correlation of serum Her-2/*neu* ECD levels with pHER-2/*neu* status

**Figure 3:**
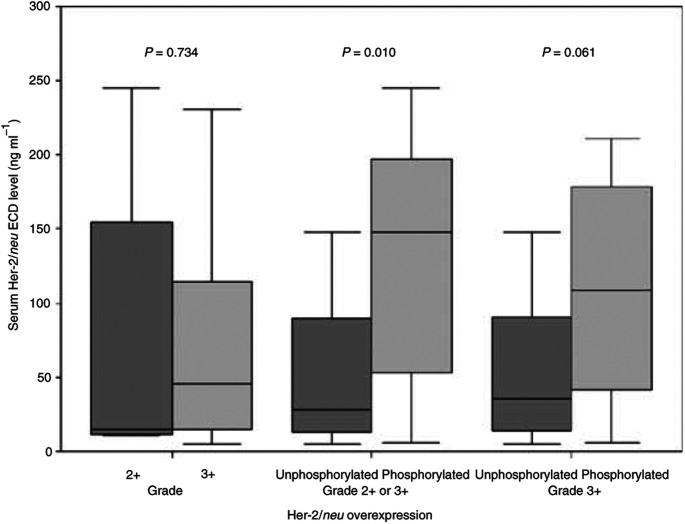
Serum Her-2/*neu* ECD levels are depicted in accordance to Her-2/*neu* overexpression (grade 2+ *vs* 3+) and Her-2/*neu* phosphorylation status of tumours (extreme outliers not depicted).

Patients with Her-2/*neu* phosphorylation presented with significantly higher serum Her-2/*neu* ECD levels before initiation of trastuzumab-based treatment (median 148.2, range 6.1–510.0 ng ml^−1^) as compared to patients without Her-2/*neu* phosphorylation (median 28.5, range 5.2–6076.2 ng ml^−1^; Mann–Whitney test: *P*=0.010, Figure 3

).

Serum Her-2/*neu* ECD levels have also been reported to correlate with the intensity of immunohistological Her-2/*neu* overexpression–with lower values observed in patients with grade 2+ Her-2/*neu*-positive tumors–and to correlate with tumour mass (Leitzel *et al*, 1992; Krainer *et al*, 1997; Kostler *et al*, manuscript submitted). In our patient collective, serum ECD levels (median, range) also tended to be higher in patients with grade 3+ Her-2/*neu* overexpressing tumours (45.2, 5.2–6076.2 ng ml^−1^) than in patients with grade 2+ Her-2/*neu* overexpressing tumours (15.4, 11.2–245.4 ng ml^−1^), but without statistical significance (*P*=0.734). Therefore, we also performed a correlation between serum Her-2/*neu* ECD levels and pHer-2/*neu* expression in the subset of grade 3+ Her-2/*neu* overexpressing tumours. In this subset, pHer-2/*neu* expressing cancers were again found to have higher median (range) serum ECD values of 108.4 (6.1–510.0) ng ml^−1^, as compared to patients without pHer-2/*n*eu expression (35.9, 5.2–6076.2 ng ml^−1^). However, this difference was not significant (*P*=0.061), presumably because of the small sample size. Figure 3 depicts serum Her-2/*neu* ECD levels according to Her-2/*neu* overexpression and phosphorylation status.

The association between serum Her-2/*neu* ECD levels and Her-2/*neu* phosphorylation status was adjusted by the grade of Her-2/*neu* overexpression and the presence/absence of visceral metastases in a multiple analysis of variance (ANOVA) with normalised Her-2/*neu* ECD as criterion variable. As shown in Table 2

**Table 2:** Multiple analysis of variance (ANOVA) applied to Her-2/*neu* ECD as normalised criterion variable

		**Median (95% CI) serum ECD (ng ml^−1^)**	***P*-value**
Tyr1248 Her-2/*neu*	Unphosphorylated	18.5 (9.3–36.7)	
	Phosphorylated	41.2 (17.5–97.3)	0.046
			
Visceral metastases	No	12.8 (5.0–33.1)	
	Yes	59.4 (32.4–109.0)	0.001
			
Grade of Her-2/*neu* overexpression	2+	19.0 (5.9–61.2)	
	3+	40.1 (24.8–65.0)	0.211
			
Number of organs with metastatic disease	1 or 2	27.5 (13.0–58.3)	
	More than 2	27.7 (12.8–59.9)	0.986

, patients with Her-2/*neu* phosphorylation had an adjusted median serum Her-2/*neu* ECD concentration of 41.2 (95% CI 17.5–97.3) ng ml^−1^, which was significantly higher as compared to patients without Her-2/*neu* phosphorylation having an adjusted median serum ECD level of 18.5 (95% CI 9.3–36.7) ng ml^−1^ (*P*=0.046). The adjusted median serum Her-2/*neu* ECD concentration of patients with visceral metastases amounted to 59.4 (95% CI 32.4–109.0) ng ml^−1^ in comparison to patients without visceral metastases having 12.8 (95% CI 5.0–33.1) ng ml^−1^ (*P*=0.001). The grade of Her-2/*neu* overexpression and the number of sites with metastatic disease did not show a significant correlation with serum Her-2/*neu* ECD levels.

### Correlation of pHer-2/*neu* status, histopathological and patients’ characteristics with response to trastuzumab-based treatment

Both objective response rates–seven out of 15 (47%) *vs* 12 out of 35 (34%, *P*=0.197)–and rates of clinical benefit–12 out of 15 (80%) *vs* 26 out of 47 (55%, *P*=0.129)–to trastuzumab-based treatment tended to be higher in patients with tumours exhibiting tyr1248-phosphorylated Her-2/*neu* as compared to those without pHer-2/*neu* expression.

Multiple logistic regression analyses were performed to evaluate the predictive role of Her-2/*neu* tyr1248 phosphorylation status upon response and benefit from trastuzumab-based treatment. In all analyses, grade of Her-2/*neu* overexpression, patient age, recurrence-free interval, anthracycline pretreatment, number of prior chemotherapeutic regimens for metastatic disease, ER and PgR status, Karnofsky's performance index, presence or absence of visceral metastases, number of organs involved by metastatic disease, type of treatment (single-agent trastuzumab *vs* combination with chemotherapy) and baseline serum Her-2/*neu* ECD levels were entered as variates.

In univariate analysis, none of the variates mentioned above significantly predicted response with significance levels below 10%. Her-2/*neu* phosphorylation status was the only variate showing a trend to predict benefit (for descriptive values see Table 3

**Table 3:** Clinical and histopathological predictors of response (complete or partial), clinical benefit (response or disease stabilisation), progression-free survival (PFS) and overall survival (OAS) with significance levels below 10% (*P*<0.1) in univariate and multivariate analyses RR=related risk; 95% CI=95% confidence interval

	**Variable**	**RR**	**95% CI**	***P*****-value**
*Response*				
Univariate	—	—	—	—
Multivariate	Her-2/*neu* phosphorylation	3.08	0.85–11.20	0.088

*Benefit*				
Univariate	Her-2/*neu* phosphorylation	3.23	0.81–2.97	0.098
Multivariate	—	—	—	—

*PFS*				
Univariate	Grade 3+Her-2/*neu* overexpression	0.44	0.20–0.95	0.036
	Her-2/*neu* phosphorylation	0.41	0.21–0.82	0.012
	Higher number of metastatic sites	2.09	1.21–3.63	0.009
	Good performance status	0.61	0.35–1.05	0.074
Multivariate	Grade 3+Her-2/*neu* overexpression	0.34	0.12–0.93	0.037
	Presence of visceral metastases	0.38	0.17–0.86	0.020
	Her-2/*neu* phosphorylation	0.38	0.18–0.81	0.012
	Higher number of metastatic sites	2.66	1.33–5.32	0.006

*OAS*				
Univariate	Grade 3+Her-2/*neu* overexpression	0.11	0.04–0.27	<0.001
	Serum Her-2/*neu* at baseline	1.00	1.00–1.00	0.076
	Higher number of metastatic sites	4.71	2.10–10.56	<0.001
	Good performance status	0.28	0.13–0.60	0.001
Multivariate	Higher number of metastatic sites	3.79	1.55–9.26	0.003
	Grade 3+Her-2/*neu* overexpression	0.15	0.05–0.48	0.001

).

In multivariate analysis, no significant predictors of response to trastuzumab-based treatment were identified; again, Her-2/*neu* phosphorylation status was the only covariate that revealed a trend to significant prediction of response to trastuzumab-based treatment, whereas no predictors of clinical benefit were identified in multivariate analysis (Table 3).

### Correlation of pHer-2/*neu* status, histopathological and patients’ characteristics with progression-free and overall survival from trastuzumab-based treatment

**Figure 4:**
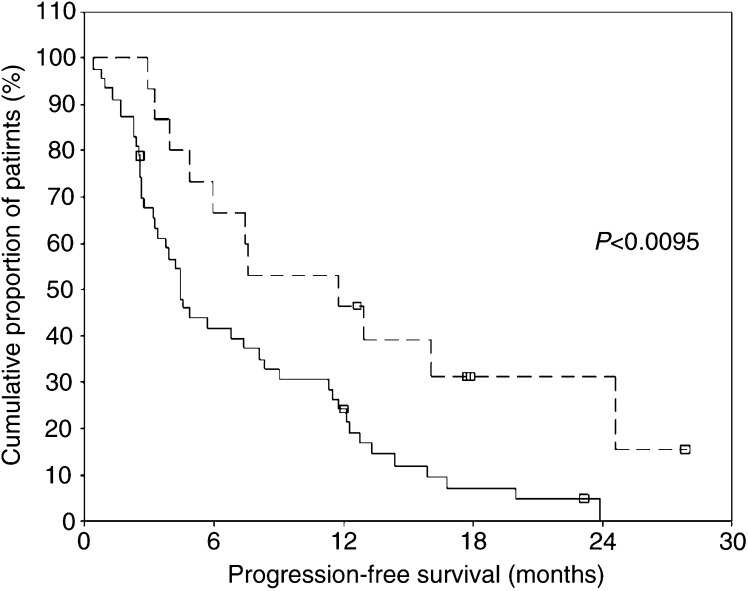
Progression-free survival in patients receiving trastuzumab-based treatment for Her-2/*neu* overexpressing metastatic breast cancer in accordance to activation status of Her-2/*neu*. Full line: unphosphorylated Her-2/*neu*, dotted line: phosphorylated Her-2/*neu* (log-rank test *P*<0.0095).

In patients with tyr1248 Her-2/*neu* phosphorylation, the median (95% CI) progression-free survival calculated from survival curves was 11.7 (95% CI 5.2–18.3) months and thus significantly longer as compared to the median (95% CI) progression-free survival of 4.5 (3.4–5.6) months observed in patients without pHer-2/*neu* staining tumours (log-rank test: *P*<0.0095). Figure 4

depicts progression-free survival curves according to Her-2/*neu* phosphorylation status. Median progression-free survival (4.4, 95% CI 2.8–6.0 months) of patients with Her-2/*neu* ECD levels below 15 ng ml^−1^ did not differ significantly from progression-free survival of patients with Her-2/n*eu* ECD above 15 ng ml^−1^ (6.7, 95% CI 2.3–11.2 months, log-rank test: *P*=0.129).

Univariate Cox regression analysis revealed that grade 3+ Her-2/*neu* overexpression, presence of Her-2/*neu* phosphorylation and low number of organs involved by metastatic disease were significant predictors of longer progression-free survival, whereas a good performance status showed a trend to a reduced risk of progression (descriptive values are depicted in Table 3). Multivariate Cox regression analysis showed that grade 3+ Her-2/*neu* overexpression, presence of visceral metastases and Her-2/*neu* tyr1248 phosphorylation were independent predictors of longer progression-free survival, whereas a higher number of organs affected by metastatic disease represented a significant adverse predictor of progression-free survival (Table 3).

Median (95% CI) overall survival in patients without pHer-2/*neu* expression was 23.8 (18.1–29.5) months and did not differ significantly from overall survival observed in patients with pHer-2/*neu* expressing tumours, which had not been reached at the time of these analyses (log-rank test: *P*=0.6842). Likewise, median overall survival of patients with Her-2/*neu* ECD levels below 15 ng ml^−1^ was not reached during the observation period. Patients with Her-2/*neu* ECD levels above 15 ng ml^−1^ had a median overall survival of 19.6 (95% CI 13.2–26.6 months, log-rank test: *P*=0.143).

In univariate Cox regression analysis for overall survival grade 3+ Her-2/*neu* overexpression, a low number of organs affected by metastatic disease and good performance status were significant predictors for longer overall survival, whereas serum Her-2/*neu* ECD levels observed at baseline did not cause significant effects (descriptive values are depicted in Table 3). In multivariate analyses, a low number of organs affected by metastatic disease and grade 3+ Her-2/*neu* overexpression yielded a reduced risk of death within the observation period (Table 3).

## DISCUSSION

The present investigation was performed to evaluate a potential correlation of tyrosine phosphorylated Her-2/*neu* with cleavage of its ECD in breast cancer patients, and to determine the clinical consequences of Her-2/*neu* activation with respect to the efficacy of trastuzumab-based treatment. We here report that, in Her-2/*neu* overexpressing tumours, increased serum Her-2/*neu* ECD levels are indeed associated with active Her-2/*neu* tyrosine kinase signalling, as represented by Tyr1248-phosphorylated Her-2/*neu*. Our findings strongly support the clinical relevance of previous *in vitro* observations, which have demonstrated that spontaneous proteolytic cleavage of the ECD represents a ligand-independent activation mechanism of Her-2/*neu* (Molina *et al*, 2001).

In this context, it is interesting to note that the detection of increased serum levels of the Her-2/*neu* ECD has been associated with increased resistance to endocrine therapy in Her-2/*neu* overexpressing metastatic breast cancer (Lipton *et al*, 2002), thereby suggesting that resistance to antihormonal agents was associated with active Her-2/*neu*-dependent signalling. On the other hand, serum Her-2/*neu* ECD levels have been associated with increased response rates and prolonged progression-free survival to subsequent trastuzumab-based treatment (Esteva *et al*, 2002; Kostler *et al*, manuscript submitted). It can, therefore, be hypothesised that not only the mere Her-2/*neu* overexpression, but also its activation status are crucial for susceptibility to the biological effects of trastuzumab *in vivo*.

Based on these assumptions, we have determined the impact of the Her-2/*neu* phosphorylation on the clinical outcome of patients with Her-2/*neu* overexpressing metastatic breast cancer receiving trastuzumab-based treatment. In the present analysis, we have found a trend towards higher response rates and higher rates of clinical benefit in patients with activated Her-2/*neu*. Notably, progression-free survival to trastuzumab-based treatment was more than doubled in patients with tumours exhibiting activated Her-2/*neu* as compared to patients lacking tyrosine-phosphorylated Her-2/*neu*. Whether this observation can be attributed to an increased and prolonged sensitivity to trastuzumab-based treatment or represents an intrinsic biologic characteristic of tumours exhibiting tyr1248 phosphorylation of Her-2/*neu* remains to be determined in future prospective trials evaluating the predictive value of pHer-2/*neu* for various forms of treatment.

In addition, the absence of Tyr1248 phosphorylation does not necessarily preclude Her-2/*neu*-dependent signalling: although Her-2/*neu* activation is thought to be a hierarchical event in which Tyr1248 is the ultimate C-terminal site to be phosphorylated before downstream signalling occurs (Akiyama *et al*, 1991; Harder *et al*, 1994), it is conceivable that interactions of other phosphorylated (non)tyrosine residues with heterodimerisation partners resulting in heterodimerisation partner-dependent secondary signalling (Graus-Porta *et al*, 1997; [Bibr bib21][Bibr bib22]; Ouyang *et al*, 2001) could also be targeted by trastuzumab. The characterisation these activation states, of stimulatory and inhibitory interactions and their correlation with the clinical effects of trastuzumab will clearly have a profound effect on our biologic understanding of receptor signalling and ultimately refine targeted therapies.
